# Racial and Gender Differences in Mindfulness, Engaged Living, and Psychological Inflexibility Within a Hawaiʻi-Based College Sample

**DOI:** 10.3390/bs16061010

**Published:** 2026-06-16

**Authors:** Lisa Nakamura, Samuel D. Spencer, Duckhyun Jo, Fumiaki Hamagami, Earl S. Hishinuma, Cerila C. Rapadas, Callum T. Henwood, Akihiko Masuda

**Affiliations:** 1 Department of Psychology, University of Hawaiʻi at Mānoa, 2530 Dole Street, Sakamaki C400, Honolulu, HI 96822, USA; lisa.nakamura001@umb.edu (L.N.); samuel.spencer@unt.edu (S.D.S.); djo3@central.uh.edu (D.J.); cerilar@hawaii.edu (C.C.R.); callumt@hawaii.edu (C.T.H.); 2 Department of Psychology, University of Massachusetts Boston, 100 Morrissey Blvd, Boston, MA 02125, USA; 3 Department of Psychology, University of North Texas, Terrill Hall, 1611 W Mulberry St., Denton, TX 76201, USA; 4 Department of Psychology, University of Houston, Heyne Building, Room 126, Houston, TX 77204, USA; 5 Department of Psychiatry, John A. Burns School of Medicine, University of Hawaiʻi at Mānoa, 651 Ilalo Street, Honolulu, HI 96813, USA

**Keywords:** college students, mindfulness, engaged living, psychological inflexibility, experiential avoidance, gender, race, ethnicity

## Abstract

Objective: Extending prior psychometric work with a Hawaiʻi-based college sample, this secondary cross-sectional study examined racial and gender group differences in mindfulness, engaged living, and psychological inflexibility within that same Hawaiʻi-based sample. Methods: Consistent with the grouping strategy used in that previous psychometric work, participants were categorized into 402 Asian American (263 women, 139 men), 260 White American (199 women, 61 men), and 406 combined All Others (301 women, 105 men) students, all recruited from a four-year public university in Hawaiʻi. After informed consent, participants voluntarily and anonymously completed an online survey containing self-report measures of interest. Results: Asian American students reported lower scores on measures of observing, describing, and non-judging facets of mindfulness than White American students. Men reported higher describing, non-distractibility, non-judging, and non-reacting scores than women, and White American men showed higher non-autopiloting than White American women. For engaged living and psychological inflexibility, White American students reported greater life fulfillment and lower psychological inflexibility than Asian American and All Others groups. Asian American women reported lower recognizing-values scores than White American women, and women overall endorsed greater psychological inflexibility than men. These differences, although statistically significant, were generally small and did not appear to be clinically meaningful. Associations among study variables as well as their roles in psychological distress were also examined within each racial group. Conclusions: Keeping several notable limitations in mind, this secondary cross-sectional investigation provided a preliminary examination of mindfulness, engaged living, and psychological inflexibility across racial and gender group categories, using methodological and psychometric decisions tailored to the present Hawaiʻi-based sample. We hope that our preliminary findings encourage further investigation in this domain.

## 1. Introduction

Today, mindfulness- and acceptance-based practices (Hayes et al., 2004; Masuda, 2014) have been widely integrated into diverse applied settings, including counseling and psychotherapy (Campbell et al., 2018; Hayes et al., 2012a; Hilert & Tirado, 2019). From the perspective of culturally responsive evidence-based practice (Haynes et al., 2018; Hays, 2009), this proliferation parallels the development and examination of self-report instruments designed to assess the key psychological processes targeted by these approaches. Notable exemplars of such assessment instruments are the *Five Facet Mindfulness Questionnaire* (FFMQ; Baer et al., 2008) for mindfulness, the *Engaged Living Scale* (ELS; Trompetter et al., 2013) for valued-based and engaged living, and the *Acceptance and Action Questionnaire-II* (AAQ-II; Bond et al., 2011) for psychological inflexibility. It is important to note at the outset the ongoing psychometric concerns regarding the AAQ-II as a measure of psychological inflexibility, which are discussed in greater detail below.

Within this applied context in the U.S., and with growing attention to multiculturalism and culturally responsive practice, practitioners and researchers have increasingly examined and validated mindfulness- and acceptance-informed measures for underrepresented populations, including racial minority groups (e.g., Edwards & Vowles, 2020; Morgan et al., 2014; Spencer et al., 2022). Building on this line of inquiry, the present secondary cross-sectional study compared the facets of the FFMQ, ELS, and AAQ-II across gender (women, men) and race (Asian Americans, White Americans, and a combined All Others group) within a Hawaiʻi-based college sample (*N* = 1068). To ensure the legitimacy of group comparisons in race and gender with the present Hawaiʻi-based sample (see Putnick & Bornstein, 2016 for a detailed discussion on this topic), the present analyses were based on the findings of a psychometric validation/measurement invariance study that was previously conducted for the FFMQ, ELS, and AAQ-II with the same Hawaiʻi-based college sample (i.e., Spencer et al., 2022). From a larger applied perspective, examining data from a Hawaiʻi-based sample is particularly suitable for this type of investigation, as the state’s racial diversity reflects projected demographic trends in the U.S. population (U.S. Census Bureau, 2019).

### 1.1. Five Facet Mindfulness Questionnaire (FFMQ)

The 39-item Five Facet Mindfulness Questionnaire (FFMQ; Baer et al., 2006, 2008) is the most widely used self-report measure of mindfulness (Baer, 2019; Carpenter et al., 2019). Grounded in conceptualizations of mindfulness as an open, nonjudgmental awareness of internal experiences and a capacity for attentional regulation (Baer, 2018; Hayes et al., 2011), the FFMQ assesses mindfulness across five interrelated yet distinct facets: *observing* (8 items; noticing or attending to experience), *describing* (8 items; labeling emotional states with words), *acting with awareness* (8 items), *non-judging of inner experience* (8 items), and *non-reactivity to inner experience* (7 items). Research has consistently shown that FFMQ scores are positively associated with indices of salutary psychological processes, including psychological well-being, emotional intelligence, and self-compassion (Baer et al., 2006, 2008; Williams et al., 2014). Conversely, FFMQ scores tend to be negatively associated with psychological symptoms, such as depression, anxiety, and stress (Carpenter et al., 2019; Jo et al., 2025), as well as maladaptive processes including experiential avoidance, thought suppression, and neuroticism (Baer et al., 2006, 2008). Baer (2019) further noted that among the five facets, *non-judging* and *non-reactivity* appear particularly relevant to mental health outcomes.

Of particular relevance to the present study, Spencer et al. (2022) previously examined the psychometric properties of the FFMQ in the present Hawaiʻi-based college sample (*N* = 1102). Their analyses indicated that a six-factor model provided a better fit than the original five-factor structure. Four of the six factors corresponded to the original *observing*, *describing*, *non-reactivity*, and *non-judging* facets, although three items were removed from the describing facet (items 12, 16, and 22). The remaining two factors, *non-distractibility* (4 items; the extent to which individuals sustain contact with present-moment experience) and *non-autopilot* (4 items; mindful engagement in daily activities), emerged from the original *acting with awareness* subscale.

### 1.2. Engaged Living Scale (ELS)

The ELS (Trompetter et al., 2013) is a 16-item self-report measure developed to assess engaged living, which is often defined as a salutary psychological process of clarifying personal values and engaging in values-consistent actions. The original validation study found a two-factor model, including *valued living* (10 items; recognizing personal values and engaging in values-consistent actions) and *life fulfillment* (6 items; having a sense of fulfillment in life) subscales. Consistent with mindfulness- and acceptance-based theories (Hayes et al., 2012b; Segal et al., 2013), the original validation study also found that the scores of ELS were positively associated with theoretically related constructs, including psychological well-being, acceptance, and mindfulness, and inversely correlated with a range of distress variables, such as anxiety and depression (Trompetter et al., 2013).

For the present Hawaiʻi-based sample, Spencer et al. (2022) identified that the optimal fitting model possessed a four-factor structure, deviating from the originally suggested two-factor model. More specifically, three new factors emerged from the *valued living* facet from the original ELS study, and these were termed *recognizing values* (4 items; being aware of one’s values), *clarity in life direction* (3 items; having a clear life direction), and *value–action congruence* (2 items; having consistency between values and actions). The fourth factor was identical to the *life fulfillment* of the original ELS, except for one item (i.e., item 10) from the original *valued living* being added to the fourth facet. In Spencer et al. (2022), the fourth facet continued to be named *life fulfillment* (7 items).

### 1.3. Acceptance and Action Questionnaire-II (AAQ-II)

The AAQ-II (Bond et al., 2011) is a 7-item self-report measure of psychological inflexibility, which is theorized as a collection of maladaptive efforts to regulate unwanted internal events at the expense of values-based living. Among samples of predominantly European or White American participants (e.g., Bond et al., 2011; Fledderus et al., 2012), scores of the AAQ-II have consistently demonstrated a unidimensional factor structure. Bond et al. (2011) also found that psychological inflexibility was positively associated with depression, anxiety, stress, and psychological distress, and inversely associated with psychological well-being and mindfulness. Particularly relevant to the present study, in the aforementioned sample from Hawaiʻi, Spencer et al. (2022) found that the most suitable factor structure for the AAQ-II consisted of a unidimensional model along with theoretically consistent residual correlations among item pairs.

Importantly, the use of the AAQ-II as a measure of psychological inflexibility requires particular caution in light of recent psychometric and validation work on the instrument. As noted in recent scholarship (Cherry et al., 2021; Doorley et al., 2020), concerns have been raised regarding its construct validity, with evidence indicating that AAQ-II scores may reflect general psychological distress or neuroticism more so than psychological inflexibility itself (Tyndall et al., 2019). Nevertheless, when the data collection for the present study began in 2018 (see Spencer et al., 2022), the literature questioning the AAQ-II was only beginning to develop (e.g., Rochefort et al., 2018; Wolgast, 2014), and alternative measures of psychological inflexibility were not yet readily available. Accordingly, the present study reports AAQ-II findings with additional caution.

### 1.4. Measurement Invariance and Data of FFMQ, ELS, and AAQ-II by Race and Gender

As noted above, and particularly relevant to the present study, Spencer et al. (2022) also evaluated measurement invariance (Putnick & Bornstein, 2016) for the FFMQ, ELS, and AAQ-II across race and gender using the present Hawaiʻi-based college sample. More specifically, given the sample size and demographic distribution, Spencer et al. (2022) categorized participants into Asian, White, and All Others racial groups, and into women and men for gender. Their analyses provided preliminary evidence that mindfulness (FFMQ), engaged living (ELS), and psychological inflexibility (AAQ-II) were invariant across these groups at the configural, metric, scalar, and strict levels. In other words, these findings support the appropriateness of conducting race and gender comparisons using these measures within the present Hawaiʻi-based sample.

### 1.5. Present Study

Following previous psychometric and measurement invariance findings with the present Hawaiʻi-based college sample (Spencer et al., 2022), the purpose of this secondary cross-sectional study was to examine and compare FFMQ, ELS, and AAQ-II scores across pre-specified race and gender categories within the same dataset. More specifically, in the present study, participants were categorized into Asian American, White American, and All Others racial groups, and into women and men for gender. Although driven primarily by psychometric and statistical considerations, it is important to acknowledge that aggregating non-Asian American and non-White American individuals into an arbitrarily created single category, the All Others group, fails to provide meaningful or conceptually coherent advancement for theory or practice in diversity psychology and well-being. Nevertheless, presenting these findings may still be useful as extremely preliminary data to guide future investigations focused on the specific groups (e.g., multiracial individuals, Native Hawaiian participants) that were combined within this category. It is also important to recognize that limiting the analysis of gender to a binary category constrains the ability to consider intersectional experiences and experiences of gender expansive individuals.

Given the limited existing literature, no a priori hypotheses were proposed; instead, exploratory analyses were conducted to examine potential group differences in FFMQ, ELS, and AAQ-II scores. In addition, as a further exploratory component, we examined associations among the study variables and their differential contributions to psychological distress within each racial group.

## 2. Method

### 2.1. Participants

As detailed in Spencer et al. (2022), participants in the present investigation were drawn from a Hawaiʻi-based college sample (*N* = 1102) recruited from undergraduate psychology courses at a large public university on Oʻahu, Hawaiʻi. The original sample included 780 women, 317 men, and 5 individuals who identified their gender as “Other.” The study did not assess whether participants identified as cisgender or transgender. Because this latter group was too small for meaningful group comparison, these participants were excluded. The limitations of and implications for excluding participants who identify their gender as other from group comparisons are further discussed in the discussion section. An additional 17 participants (8 men, 9 women) were removed for not reporting their sexual orientation, and 12 participants (4 men, 8 women) were excluded for not reporting their age. The final analytic sample consisted of 1068 participants.

The average age of the final sample was 20.32 years (*SD* = 4.06; range = 18–55). The most prevalent self-identified racial and ethnic groups were Asian American (*n* = 402), White American (non-Hispanic; *n* = 260), and multiracial (*n* = 259), followed by Latine (*n* = 62) and Native Hawaiian (*n* = 33). Consistent with the grouping strategy used in Spencer et al. (2022), participants were categorized into Asian American (*n* = 402; 263 women, 139 men), White American (*n* = 260; 199 women, 61 men), and All Others (*n* = 406; 301 women, 105 men).

The Asian American group reflected substantial ethnic diversity: Filipino (25.6%), Japanese (22.6%), Chinese (13.7%), Korean (5.5%), and other or mixed Asian backgrounds (32.6%). Regarding sexual orientation, 47 Asian Americans (32 women, 15 men), 46 White Americans (42 women, 4 men), and 78 All Others participants (59 women, 19 men) identified as gay or lesbian, bisexual, or other.

### 2.2. Study Procedures and Measures

All study procedures were approved by the Institutional Review Board at the senior author’s affiliated academic institution. Participants were recruited through an online research participation system (i.e., SONA) maintained by the Department of Psychology at the same institution. After providing informed consent online, participants anonymously and voluntarily completed a survey that included a demographic questionnaire and the self-report measures described below. Psychometric information for the measures used in the present investigation, validated within this sample, is detailed in Spencer et al. (2022).

### 2.3. Demographic Form

The demographic form consisted of questions pertaining to gender, age, race, and sexual orientation. Gender was dummy-coded as 0 (man) or 1 (woman). Age was coded as a continuous variable. Race contained the following categories: Asian, Black, Latine, Pacific Islander, Native American, Native Hawaiian, White, Multiracial, or Other. Participants were asked to select the racial category that best described them. Finally, sexual orientation consisted of the following categories: heterosexual, gay or lesbian, bisexual, or other.^1^

### 2.4. Five Facet Mindfulness Questionnaire (FFMQ)

The validated measure of the FFMQ (Spencer et al., 2022) used in the present study consists of 36 items measuring six facets of mindfulness, including *observing*, *describing*, *non-reactivity*, *non-judging*, *non-distractibility*, and *non-autopilot.* Each statement is evaluated on a Likert scale, ranging from 1 (*never or very rarely true)* to 5 (*very often or always true).* Higher FFMQ scores indicate greater levels of mindfulness.

### 2.5. Engaged Living Scale (ELS)

The validated measure of the ELS (Spencer et al., 2022) consists of 16 items measuring four facets of values-based living, including *recognizing values*, *clarity in life direction*, *value–action congruence*, and *life fulfillment.* Each statement is assessed on a Likert scale, ranging from 1 (*completely disagree)* to 5 (*completely agree).* Higher scores reflect greater levels of engaged living.

### 2.6. Acceptance and Action Questionnaire-II (AAQ-II)

The validated measure of the AAQ-II (Spencer et al., 2022) consists of 7 items measuring psychological inflexibility. Items are rated on a Likert-scale ranging from 1 (*never true*) to 7 (*always true).* Higher scores on the AAQ-II correspond to greater levels of psychological inflexibility.

### 2.7. General Health Questionnaire (GHQ)

Psychological distress was measured via the General Health Questionnaire-12 (GHQ-12; Goldberg et al., 1997). This is a 12-item self-report measure of current general psychological distress (e.g., “Have you recently felt constantly under strain?”). Research has indicated that a unidimensional factor best captures the structure of the GHQ-12, particularly when accounting for method effects linked to negatively worded items (Molina et al., 2014). The GHQ-12 utilizes a 4-point Likert scale ranging from 0 (*not at all*) to 3 (*much more than usual*). Higher scores reflect greater general psychological distress.

### 2.8. Data Analytic Plan

In the present study, we used IBM SPSS Statistics (version 29) for data analyses. We selected the following analytic approaches for their clarity, interpretability, and/or consistency with prior literature using Hawai‘i-based samples (e.g., Kam et al., 2019; Nakamura et al., 2022). First, we performed logistic regression tests to examine whether the distribution of gender and sexual orientation differed across racial groups. We then conducted a series of 2 (gender; women and men) × 3 (race; Asian American, White American, and All Others) analyses of co-variance (ANCOVA) for the scores of FFMQ, ELS, and AAQ-II while controlling for age and sexual orientation (0 = heterosexual; 1 = gay or lesbian, bisexual, or other; LGBTQ+) as covariates. ANCOVA assumptions were met, including homogeneity of variance, homogeneity of regression slopes, and approximate normality of residuals. Given the large sample size (*N* > 1000), the analyses were considered robust to minor deviations from normality. The examination of significant effects for racial group, gender, or the interaction of race and gender was followed by pairwise comparisons with a Bonferroni correction to maintain the overall alpha at 0.05.^2^

Subsequently, we conducted three hierarchical regression analyses (Aiken & West, 1991) separately for each racial group to examine the relative contributions of the facets of mindfulness (FFMQ), engaged living (ELS), and psychological inflexibility (AAQ-II) to psychological distress (GHQ). Visual inspection of residual plots, Q-Q plots, and component-plus-residual plots confirmed that the assumptions of linearity, normality, homoscedasticity, and error independence were sufficiently met across the models. Furthermore, multicollinearity diagnostics indicated that all variance inflation factors (VIFs) remained below 3. For each racial group, gender, age, and sexual orientation were entered in the first step to predict psychological distress, followed by the facets of mindfulness, engaged living, and psychological inflexibility in the second step.

## 3. Results

Results revealed that the proportion of women in the White American group (Wald *χ*^2^ = 10.38, *e^β^* = 1.77, *p* = 0.001) and the All Others group (Wald *χ*^2^ = 7.39, *e^β^* = 1.51, *p* = 0.007) were significantly greater than that of the Asian American group. Similarly, the proportions of LGBTQ+ identifying individuals in the White American group (Wald *χ*^2^ = 5.12, *e^β^* = 1.66, *p* = 0.024) and the All Others group (Wald *χ*^2^ = 9.34, *e^β^* = 1.84, *p* = 0.002) were significantly greater than that of the Asian American group. In gender and sexual orientation distributions, we did not find other racial group differences.

### 3.1. Facets of Mindfulness

Table 1 shows the means and standard deviations of the study variables by participants’ race and by gender. Results of ANCOVAs revealed a main effect of race in the *observing* [*F*(2, 1060) = 7.27, *p* = <0.001], *describing* [*F*(2, 1060) = 4.33, *p* = 0.013], and *non-judging* [*F*(2, 1060) = 3.57, *p* = 0.029] facets of mindfulness (see Table 2). Pairwise comparisons showed that the Asian American group showed significantly lower levels of *observing* [*d* = −0.29, 95% CI (−3.43, −0.75), *p* < 0.001], *describing* [*d* = −0.23, 95% CI (−1.99, −0.20), *p* = 0.010], and *non-judging* [*d* = −0.21, 95% CI (−3.03, −0.14), *p* = 0.026] facets of mindfulness than the White American group.

With respect to gender differences, our ANCOVA results revealed a main effect of gender in *describing* [*F*(1, 1060) = 5.00, *p* = 0.026], *non-distractibility* [*F*(1, 1060) = 10.48, *p* = 0.001], *non-autopiloting* [*F*(1, 1060) = 6.23, *p* = 0.013], *non-judging* [*F*(1, 1060) = 4.48, *p* = 0.035], and *non-reacting* [*F*(1, 1060) = 15.80, *p* = <0.001] facets of mindfulness. Pairwise comparisons indicated that men showed greater levels of *describing* [*d* = 0.15, 95% CI (0.08, 1.24), *p* = 0.026], *non-distractibility* [*d* = 0.22, 95% CI (0.33, 1.33), *p* < 0.001], *non-autopiloting* [*d* = 0.17, 95% CI (0.13, 1.05), *p* = 0.013], *non-judging* [*d* = 0.14, 95% CI (0.07, 1.94), *p* = 0.035], and *non-reacting* [*d* = 0.27, 95% CI (0.54, 1.59), *p* < 0.001] facets of mindfulness than women. Finally, we found a significant race x gender interaction effect in the *non-autopiloting* facet of mindfulness, *F*(2, 1060) = 3.47, *p* = 0.032. Pairwise comparisons revealed that only among the White American group, men demonstrated a significantly higher level of *non-autopiloting* than women [*d* = 0.42, 95% CI (0.44, 2.35), *p* = 0.004].

### 3.2. Facets of Engaged Living

As shown in Table 3, results of ANCOVAs revealed a main effect of race in the *life-fulfillment* facet of engaged living, *F*(2, 1060) = 4.47, *p* = 0.012. Pairwise comparisons showed that the White American group showed significantly greater *life fulfillment* than the Asian American [*d* = 0.21, 95% CI (0.12, 2.60), *p* = 0.027] and All Others [*d* = 0.22, 95% CI (0.21, 2.77), *p* = 0.016] groups. Results of ANCOVAs also revealed a significant race × gender interaction effect in the *recognizing values* facet of engaged living, *F*(2, 1060) = 3.29, *p* = 0.038. Pairwise comparisons revealed that Asian American women showed a significantly lower level of *recognizing values* than women in the White American [*d* = −0.24, 95% CI (−1.30, −0.05), *p* = 0.029] and All Others [*d* = −0.25, 95% CI (−1.25, −0.13), *p* = 0.009] groups. Our ANCOVA results did not reveal any main effect of gender.

### 3.3. Psychological Inflexibility

Finally, results of an ANCOVA revealed the main effects of race [*F*(2, 1060) = 8.21, *p* < 0.001] and gender [*F*(1, 1060) = 15.90, *p* < 0.001] in *psychological inflexibility* (see Table 4). Pairwise comparisons showed that the White American group had a lower degree of *psychological inflexibility* than the Asian American [*d* = −0.32, 95% CI (−5.50, −1.42), *p* < 0.001] and All Others [*d* = −0.22, 95% CI (−4.46, −0.28), *p* = 0.020] groups, and that women endorsed a greater degree of *psychological inflexibility* than men [*d* = 0.27, 95% CI (1.36, 3.98), *p* < 0.001].

### 3.4. The Role of Sexual Orientation in Mindfulness, Engaged Living, and Psychological Inflexibility

In ANCOVAs reported above, we also found sexual orientation to be a significant predictor for many of the dependent variables of interest. These include the *observing* [*F*(1, 1060) = 16.25, *p* < 0.001], *describing* [*F*(1, 1060) = 9.09, *p* = 0.003], *non-distractibility* [*F*(1, 1060) = 21.82, *p* < 0.001], *non-autopiloting* [*F*(1, 1060) = 23.02, *p* < 0.001], and *non-judging* [*F*(1, 1060) = 14.14, *p* < 0.001] facets of mindfulness as well as the *recognizing values* [*F*(1, 1060) = 11.98, *p* < 0.001], *value–action congruence* [*F*(1, 1060) = 4.54, *p* = 0.033], and *life fulfillment* [*F*(1, 1060) = 14.97, *p* < 0.001] facets of engaged living, and *psychological inflexibility*, [*F*(1, 1060) = 35.49, *p* < 0.001]. More specifically, with respect to mindfulness, LGBTQ+ identifying individuals had greater levels of *describing* and *observing*, but showed lower levels of *non-distractibility*, *non-autopiloting*, and *non-judging* than heterosexual individuals. For engaged living, LGBTQ+ individuals showed lower levels of *recognizing values*, *value–action congruence*, and *life fulfillment* than the heterosexual group. Finally, LGBTQ+ individuals showed a significantly greater level of *psychological inflexibility* than their heterosexual counterparts.

### 3.5. Roles of Mindfulness, Engaged Living, and Psychological Inflexibility on Psychological Distress Across Racial Groups

As shown in Table 5, Table 6 and Table 7, descriptive statistics and correlations among the non-dichotomous variables included in the subsequent regression analyses are presented by racial group. Below are the results of the regression analyses for each racial group (also see Table 8).

**Asian American.** Results of the hierarchal regression revealed that after controlling for age, gender, and sexual orientation (Step 1; *R*^2^ = 0.03), *non-distractibility facet of mindfulness* (*β* = −0.11, *p* < 0.05), *recognizing value* (*β* = −0.13, *p* < 0.05), *value–action congruence* (*β* = 0.13, *p* < 0.05), *life fulfillment* (*β* = −0.13, *p* < 0.01), and *psychological inflexibility* (*β* = 0.31, *p* < 0.01) were uniquely associated with psychological distress (Step 2; *R*^2^ = 0.49). The direction of these significant findings is generally theoretically consistent with the extant literature, with the exception of the positive association between value–action congruence and psychological distress observed in the present Asian American sample. For this sample, a suppressor effect was observed for the *describing* subscale. Although it had a significant negative bivariate relationship with distress (*r* = −0.18, *p* < 0.05; Table 5), its unique relationship was positive (*r*_partial_ = 0.04, *p* > 0.05) once shared variance with the other mindfulness, engaged living, and psychological inflexibility predictors was statistically controlled.

**White American.** The hierarchal regression with the White American sample revealed that after controlling for age, gender, and sexual orientation (Step 1; *R*^2^ = 0.13), being a woman (*β* = 0.11, *p* < 0.05), *describing* (*β* = −0.13, *p* < 0.05) and *non-reactivity* (*β* = −0.12, *p* < 0.05) *facets of mindfulness*, *life fulfillment* (*β* = −0.19, *p* < 0.05), and *psychological inflexibility* (*β* = 0.19, *p* < 0.01) were uniquely associated with psychological distress (Step 2; *R*^2^ = 0.47). The direction of these significant findings is theoretically consistent with the extant literature.

**All Others Group.** Finally, the hierarchal regression with the All Others group revealed that after controlling for age, gender, and sexual orientation (Step 1; *R*^2^ = 0.02), *non-judging facet of mindfulness* (*β* = −0.21, *p* < 0.01), *life fulfillment* (*β* = −0.23, *p* < 0.01), and *psychological inflexibility* (*β* = 0.25, *p* < 0.01) were uniquely associated with psychological distress (Step 2; *R*^2^ = 0.46). Similarly to the findings of White American group, the direction of these significant findings in the All Others group is theoretically consistent with the extant literature.

## 4. Discussion

In recent years, it has become increasingly important to examine and compare psychological constructs across racial groups using psychometrically sound measures (Haynes et al., 2018; Putnick & Bornstein, 2016). In the present exploratory cross-sectional study, we examined racial and gender group differences in measures of facets of mindfulness, engaged living, and psychological inflexibility, constructs that are central to mindfulness- and acceptance-based interventions (Hayes et al., 2011; Masuda, 2014), within a Hawaiʻi-based college sample. More specifically, we employed psychometrically validated measures that were previously established for use with this Hawaiʻi-based sample (Spencer et al., 2022). These constructs were selected because they have become integrated into various applied fields today, including counseling and psychotherapy (Campbell et al., 2018; Hayes et al., 2012a; Hilert & Tirado, 2019). Worth noting here is that we only proceeded with examining group mean differences on these measures in the present study after establishing measurement invariance of these scales in a previous psychometric study (i.e., Spencer et al., 2022). This additional step to establish preliminary psychometric properties of the measures under investigation with the specific population of interest is an important yet often neglected consideration for best practices in psychological assessment, especially within diverse cultural contexts (Haynes et al., 2018, 2019).

Our results revealed that, when controlling for age and sexual orientation, the Asian American group showed significantly lower scores on measures of *observing*, *describing*, and *non-judging* facets of mindfulness than the White American group, and that women had significantly lower scores on measures of *describing*, *non-distractibility*, *non-autopiloting*, *non-judging*, and *non-reacting* facets of mindfulness than men. Within the White American group, women evidenced significantly lower scores on *non-autopiloting* than men. For the facets of engaged living and psychological flexibility, the White American group showed greater scores on a measure of *life fulfillment* as well as significantly lower scores on a measure of *psychological inflexibility* than the Asian American and All Others groups. Furthermore, Asian American women showed a significantly lower score on a measure of *recognizing values* than White American women, and women showed a greater score on a measure of *psychological inflexibility* than men. Importantly, although statistically significant, these observed differences were small or trivial in magnitude, however.

Across all racial groups, we also found that *life fulfillment* (as a facet of engaged living) and *psychological inflexibility*, the construct the AAQ-II is designed to measure, emerged as statistically significant correlates of psychological distress after controlling for key demographic variables, the facets of mindfulness, and other dimensions of engaged living. In contrast, the specific mindfulness facet(s) associated with psychological distress varied by racial group: *non-distractibility* for Asian American participants, *describing* and *non-reactivity* for White American participants, and *non-judging* for participants in the All Others group.

Furthermore, among Asian American participants, *recognizing values* and *value–action congruence* emerged as additional correlates of distress. Somewhat unexpectedly, the positive association between *value–action congruence* and distress ran counter to patterns typically reported in the literature (Hayes et al., 2011; Lin et al., 2024; Trompetter et al., 2013). Notably, however, this positive association was small in magnitude.

Taken together, our findings indicate that, for the gender and racial groups represented in this sample, observed group differences do not extend uniformly across all facets of mindfulness, engaged living, and psychological inflexibility, and that the differences that do emerge are small in magnitude. Moreover, the results suggest that the *life-fulfillment* facet of engaged living and *psychological inflexibility*, the construct the AAQ-II is intended to measure, were consistently associated with psychological distress across racial groups.

In the following sections, we discuss these group differences in greater detail for each construct. Before doing so, several considerations are important when interpreting the present findings. First, the FFMQ, ELS, and AAQ-II, the measures used in this study, were originally developed and validated primarily with White American and European samples (see Baer et al., 2008; Bond et al., 2011; Trompetter et al., 2013). As such, the constructs of mindfulness, engaged living, and psychological inflexibility may reflect the cultural practices and values of these groups more strongly than those of other racial and ethnic groups, a phenomenon referred to as construct bias (van de Vijver & Tanzer, 2004).

Relatedly, although preliminary evidence of measurement invariance for these scales was established across the racial and gender groups included in the present study (Spencer et al., 2022), it remains possible that the underlying constructs may vary qualitatively across groups in meaningful ways (Haynes et al., 2018). Measurement invariance supports the comparability of scores at the statistical level, but it does not guarantee that the psychological meaning or lived experience of these constructs is identical across cultural contexts.

Additionally, the measures used to operationalize the constructs under investigation in the present study are inherently limited by the fact that definitive operationalization of—by definition unobservable—latent constructs is an aspirational (yet perhaps unattainable) goal in psychological science (Flake et al., 2017; Meehl, 1978). Moreover, statistical relations among and between measures of purported constructs operate under principles of multifinality and equifinality, which inherently limit inferences regarding said relations (Di Plinio et al., 2024). Furthermore, findings from group-level comparisons also may not necessarily generalize to unique individuals, further limiting generalizability (Molenaar, 2004). Thus, a significant degree of intellectual humility, qualification, and consideration of counterfactuals is needed when interpreting findings from the present study (Washburn et al., 2022).

Finally, individuals’ upbringing and life experiences may shape how they respond to items on these measures (Haynes et al., 2018). For example, experiences of oppression, such as racial discrimination or heterosexism, may influence how participants interpret and endorse items on the FFMQ, ELS, and AAQ-II, potentially contributing to group differences in scale scores. With these considerations in mind, we now turn to the discussion of our findings in FFMQ, ELS, and AAQ-II scores.

### 4.1. Facets of Mindfulness

There is limited evidence examining racial and ethnic group differences in the facets of mindfulness measured by the FFMQ (e.g., Baer et al., 2008; Christopher et al., 2012). As noted above, our findings from a Hawaiʻi-based college sample revealed statistically significant, but small differences between Asian American and White American participants in measures of three of the six mindfulness facets. Although a comprehensive investigation of the mechanisms underlying these differences is beyond the scope of the present study, it is worthwhile to tentatively consider the possible psychological and sociocultural contexts in which these assessments and comparisons were made. Such reflections should be approached with caution given the exploratory nature of the current analyses, yet they may offer a useful starting point for future, more targeted research.

One possible psychological context for these small differences involves communication styles and practices (Park & Kim, 2008). Broadly speaking, compared to many European American cultural contexts, Asian American cultural contexts tend to rely more heavily on high-context communication, in which internal and external experiences (e.g., thoughts and feelings) are conveyed subtly and implicitly rather than through explicit verbalization. From this perspective, the Asian American group’s lower scores on the *describing* facet may be unsurprising, as describing or labeling internal experiences is itself an act of explicit articulation, a hallmark of low-context communication common in many European American cultures. This cultural account may also align with the findings of the Asian American group’s lower scores on the *observing* facet, given that the type of observation assessed by the FFMQ often reflects a similar process of explicit noticing (e.g., “I notice visual elements in art or nature, such as colors, shapes, texture.”).

Furthermore, the extant literature suggests that, consistent with the Asian and Asian American cultural value of emotional self-control (Kim et al., 2001; Komiya et al., 2000), Asian Americans tend to appraise their negative affect and unpleasant thoughts unfavorably. The *non-judging* facet of the FFMQ appears to assess the degree to which individuals refrain from evaluating such internal experiences, particularly negative emotions and thoughts (e.g., “I criticize myself for having irrational or inappropriate emotions”). From this perspective, cultural values emphasizing emotional self-control may help contextualize Asian Americans’ significantly lower scores on the *non-judging* facet of mindfulness relative to their White American peers.

Our results also indicated gender differences across measures of multiple facets of mindfulness. After adjusting for age and sexual orientation, women reported lower scores on measures of *describing*, *non-distractibility*, *non-judging*, and *non-reacting* compared to men, with effect sizes ranging from trivial to small, depending on the facet. Within the White American subgroup, women additionally demonstrated lower scores on a measure of *non-autopiloting*. Given that the extant literature consistently conceptualizes mindfulness as a gender-neutral construct (Baer, 2019), this pattern of gender differences may simply reflect statistical artifacts rather than meaningful psychological distinctions. However, emerging evidence also suggests that women’s lower mindfulness scores may, at least partially and to a small degree, reflect heightened self-criticism, evaluative tendencies, emotional intensity, and rumination (e.g., Johnson & Whisman, 2013), psychological domains directly assessed by several FFMQ facets. Another relevant psychosocial factor to consider is that discrepancies across studies may stem from differences in sample composition and characteristics, as earlier work relied largely on predominantly White American or European samples (Baer et al., 2008; Christopher et al., 2012). Given our findings in light of previous findings, coupled with the aforementioned measurement-related limitations of the present study, it may be worthwhile to examine additional, as-yet-unidentified factors further, such as perceived pressure to adhere to gender roles (e.g., Yarnell et al., 2019) in the context of mindfulness both at group and individual levels.

### 4.2. Facets of Engaged Living

To our knowledge, no prior study has examined gender or racial differences in measures of ELS facets (Trompetter et al., 2013). In the present Hawaiʻi-based college sample, our findings indicate statistically significant, but small racial group differences in the measure of only one facet of engaged living. Specifically, White American participants reported significantly higher scores on the measure of *life fulfillment* than both Asian American and All-Others groups. No racial group differences emerged across measures of the remaining facets of engaged living (i.e., *recognizing values*, *clarity in life direction*, and *value–action congruence*). Furthermore, no main effects of gender were observed for any of the measures of the four facets of engaged living, suggesting that scores of the measures of these facets are generally comparable across gender. However, an exception emerged for the *recognizing values* facet: Asian American women demonstrated statistically significantly lower scores, albeit small in magnitude, than women in the White American and All-Others groups.

Our findings on engaged living can be understood in light of the broader literature on life fulfillment and value. Previous research suggests that various factors are relevant to individuals’ perceived life fulfillment and recognition of personal values, including good physical health, supportive social relationships, gender roles and perceived expectation, and the fulfillment of basic psychological needs (Diener et al., 2018; Oishi et al., 2009; Schwartz & Rubel-Lifschitz, 2009) Hence, it is important to consider the extent to which the conceptualization of life fulfillment may vary across cultures. Prior research indicates that cultural contexts, such as differences between more individualistic and more collectivistic value systems, can moderate the associations between these factors (e.g., physical health, personal values, social relationships, etc.) and perceived life fulfillment (Oishi et al., 2009). Given the multicultural context of Hawaiʻi, future research would benefit from identifying the specific cultural, interpersonal, and contextual factors that may be associated with life fulfillment or recognition of personal values in Hawaiʻi-based samples at both group and individual levels.

### 4.3. Psychological Inflexibility

Our results indicated that White American students evidenced lower scores on a measure of psychological inflexibility than both Asian American and All-Others groups. This pattern differs somewhat from previous research suggesting that levels of psychological inflexibility, as measured by the AAQ-II, are generally comparable across Asian American, White American, and other racial and ethnic groups (e.g., Kam et al., 2019; Nakamura et al., 2022). As with the racial differences observed in a measure of engaged living in the present study, these group differences, although small, in psychological inflexibility warrant careful empirical and conceptual attention, especially given psychometric challenges in measuring psychological inflexibility that have been described elsewhere (Spencer & Tyndall, in press). Additionally, the extent to which recent research (much of which has been conducted since the origination of the present study) has revealed psychometric issues with the AAQ-II vis-à-vis problematic overlap with measures of neuroticism (i.e., discriminant validity) limits inferences drawn from findings of the present study (Cherry et al., 2021; Tyndall et al., 2019).

Furthermore, consistent with the extant literature (e.g., Bond et al., 2011), we found that women endorsed higher scores on the AAQ-II than men. Once again, these results highlight the need for further examination of the nature of racial, ethnic, and gender group differences reflected in AAQ-II scores, particularly in light of ongoing concerns regarding the measure’s construct validity and its potential overlap with general negative affectivity.

### 4.4. Roles of Mindfulness, Engaged Living, and Psychological Inflexibility on Distress

Finally, mindfulness, engaged living, and psychological inflexibility are of particular interest in applied settings because of their established links to behavioral health (Hayes et al., 2011). Translating this conceptual finding to the present study, we found that our measures of the *life-fulfillment* facet of engaged living and *psychological inflexibility*, or, more precisely, the construct assessed by the AAQ-II, emerged as consistent correlates of our measure of psychological distress across racial groups, even after accounting for demographic factors, mindfulness facets, and other components of engaged living. Extending prior work (Lin et al., 2024; Trompetter et al., 2013) with respect to the role of engaged living, these findings tentatively suggest that among the various dimensions of engaged living, *life fulfillment* may be particularly salient in relation to psychological distress.

As described throughout this paper, interpretation of the psychological inflexibility findings requires particular caution because this construct was assessed using the AAQ-II. As noted in recent scholarship (Cherry et al., 2021; Doorley et al., 2020), concerns have been raised regarding the measure’s construct validity, with evidence indicating that AAQ-II scores may reflect general psychological distress or neuroticism more so than psychological inflexibility (Tyndall et al., 2019). In the present study, correlations between psychological inflexibility and psychological distress were large, ranging from 0.57 to 0.62 across the three racial groups, suggesting substantial content overlap. The potential problematic conceptual and psychometric overlap between the AAQ-II and GHQ in the present study necessitates caution in the interpretation of findings, especially concerning results of exploratory hierarchical regression models that employ the AAQ-II as a predictor and GHQ as a dependent variable. Accordingly, future research is encouraged to replicate these findings using more refined measures of psychological inflexibility with Hawai‘i-based samples. These efforts are currently underway (e.g., Jo et al., 2023).

Another noteworthy finding from this set of analyses is that measures of mindfulness facets associated with psychological distress varied across racial groups in the present study: *non-distractibility* for Asian American participants, *describing* and *non-reactivity* for White American participants, and *non-judging* for those in the All Others group. The patterns observed for the White American and All Others groups align with prior research, as the existing literature has identified *the non-reactivity* and *non-judging* facets of mindfulness as particularly relevant to mental health outcomes (Baer, 2019; Carpenter et al., 2019). In contrast, it is somewhat unexpected that *non-distractibility* emerged as the only mindfulness facet uniquely associated with psychological distress among Asian American participants after accounting for other mindfulness facets, engaged-living dimensions, and psychological inflexibility. This finding for the Asian American group warrants replication, and if replicated, future research should examine potential mechanisms that might explain why non-distractibility plays a distinctive role in psychological distress within this population. Interestingly, one potential explanation for this discrepant finding is highlighted by examining the regression diagnostics, which showed a suppressor effect for specific mindfulness facets, most notably *describing*. This divergence between zero-order and partial correlations suggests that while describing internal states is bivariately associated with lower distress, its unique predictive power is suppressed by its strong conceptual overlap with global psychological inflexibility (i.e., AAQ-II) and other mindfulness facets in Asian Americans.

Yet another noteworthy finding is that among Asian American participants, measures of the *recognizing values* and *value–action congruence* facets of engaged living emerged as additional unique predictors of a measure of psychological distress, with *value–action congruence* showing a positive association with distress. This pattern runs counter to the existing literature (Jo et al., 2022; Lin et al., 2024; Trompetter et al., 2013), which typically links greater *value–action congruence* with lower psychological distress. Although the effect size in the present study was small and may potentially reflect a statistical artifact, this unexpected association warrants replication and examination for its possible conceptual and empirical relevance. Interestingly, the small and yet statistically significant association between *value–action congruence* and distress was found only in the Asian American group, and it may be worthwhile to explore the nature of value–action congruence in this group (e.g., externally imposed/culturally prescribed values vs. self-determined values) or under what circumstance this positive association is likely at the individual level regardless of the individual’s gender and race. If replicated, future research should examine the correlates or moderators that may account for this positive relationship.

### 4.5. Sexual Orientation

Although not central to our study aims, sexual minority status emerged as a significant predictor across most facets of study measures of mindfulness, engaged living, and psychological inflexibility. Specifically, LGBTQ+ identifying individuals reported higher scores on measures of *describing* and *observing* and lower levels of *non-distractibility*, *non-autopiloting*, and *non-judging* facets of mindfulness than the heterosexual group. With respect to engaged living and psychological inflexibility, LGBTQ+ individuals evidenced lower scores on measures of *recognizing values*, *value–action congruence*, and *life fulfillment*, and greater scores on measures of *psychological inflexibility*, as measured by the AAQ-II, than the heterosexual group. These patterns, although the differences were small in magnitude, are concerning and align with prior research documenting disparities in psychological processes and outcomes among sexual minority individuals (Baer et al., 2008; Bond et al., 2011; Jo et al., 2022). Collectively, these findings tentatively align with prior research indicating that members of the LGBTQ+ community may be at heightened risk for psychological distress compared with their heterosexual peers (Cochran et al., 2003; Pitman et al., 2022). Future research should examine contextual factors that may contribute to these small and yet critical disparities in outcomes among sexual minority individuals.

### 4.6. Limitations and Future Directions

The present study has several limitations. First, the findings have limited external validity because the sample was drawn from a single university in an urban area of Hawaiʻi and consisted primarily of emerging adults. Second, racial group comparisons were restricted to three categories (Asian American, White American, and All Others) due to the sample’s demographic composition and subgroup sizes. These broad categories do not adequately represent the sociocultural heterogeneity that exists within each racial and ethnic group (e.g., Holland & Palaniappan, 2012). In particular, the All Others category combines groups with considerably heterogeneous experiences of racialization, sociocultural histories, and relationships to conceptions of mindfulness, engaged living, and psychological flexibility, which may conceal meaningful between-group differences that adequate representation could allow us to detect. This limitation is especially consequential given that the All Others category subsumes Native Hawaiian participants in a study conducted within the context of Hawaiʻi, thereby perpetuating the systemic erasure of Native Hawaiian representation in psychological research (Sasa & Yellow Horse, 2022). Future research should prioritize purposeful recruitment of participants from underrepresented racial and ethnic communities. Similarly, excluding participants who identified their gender as “Other” introduces important interpretive limitations for the group comparisons conducted in this study (American Psychological Association, 2015). To support more robust and equitable analyses, future research should prioritize recruiting samples with more balanced and inclusive gender representation, with a particular focus on recruiting gender expansive individuals.

Third, although we identified group differences across race and gender in facets of mindfulness, engaged living, and psychological inflexibility, the conceptual and clinical significance of these differences remains unclear. Fourth, the present analytic approach involved multiple ANCOVAs and regression models with intercorrelated predictors, which may increase susceptibility to Type I error, multicollinearity, and suppression effects despite the use of correction procedures and diagnostic checks. As such, the current findings should be considered exploratory, and future research using more confirmatory analytic approaches is warranted.

Finally, although previous studies have suggested measurement equivalence across gender and racial groups (Spencer et al., 2022), the observed gender and racial group differences raise the possibility that the constructs of mindfulness, engaged living, and psychological inflexibility, and the measures used to assess them, may contain systematic biases that warrant careful examination in future research (Haynes et al., 2018; Masuda et al., 2020). Said another way, findings from the current study should be considered exploratory in nature, especially with regard to aforementioned challenges within operationalization and measurement of study variables and associated limitations in generalization of study findings to populations of interest.

### 4.7. Conclusions

With several notable limitations, this secondary cross-sectional investigation offers a preliminary examination of mindfulness, engaged living, and psychological inflexibility across gender and racial group categories, using methodological and psychometric decisions appropriate for the present Hawaiʻi-based sample. These findings should be interpreted cautiously, yet we hope they provide a foundation for future research that further clarifies these constructs and their relevance across diverse populations. Future work should extend these results by examining these constructs in more nuanced ways and by employing approaches that are both individually and culturally sensitive.

## Figures and Tables

**Table 1:** Means and standard deviation of study variables by race, ethnicity and gender.

	Asian American			White American			All Others			Total		
	Man (*n* = 139)	Woman (*n* = 263)	Total (*n* = 402)	Man (*n* = 61)	Woman (*n* = 199)	Total (*n* = 260)	Man (*n* = 105)	Woman (*n* = 301)	Total (*n* = 406)	Man (*n* = 305)	Woman (*n* = 763)	Total (*N* = 1068)
FFMQ Observing	27.12 (6.45)	27.48 (6.04)	27.35 (6.18)	29.57 (5.98)	29.41 (6.11)	29.45 (6.07)	27.99 (5.48)	29.01 (6.66)	28.75 (6.38)	27.91 (6.09)	28.59 (6.36)	28.39 (6.28)
FFMQ Describing	15.79 (3.88)	14.76 (4.22)	15.12 (4.13)	16.89 (3.87)	16.02 (4.06)	16.22 (4.02)	15.65 (3.72)	15.70 (4.50)	15.68 (4.31)	15.96 (3.84)	15.46 (4.32)	15.60 (4.19)
FFMQ Non-Distractibility	12.43 (3.81)	11.74 (3.59)	11.98 (3.68)	12.79 (3.72)	10.94 (3.55)	11.37 (3.66)	11.68 (3.42)	11.45 (3.72)	11.51 (3.64)	12.24 (3.67)	11.42 (3.64)	11.65 (3.67)
FFMQ Non-Autopiloting	13.01 (3.71)	13.17 (3.35)	13.12 (3.47)	14.05 (3.36)	12.40 (3.21)	12.79 (3.31)	13.29 (3.08)	12.74 (3.36)	12.88 (3.30)	13.31 (3.45)	12.80 (3.33)	12.95 (3.37)
FFMQ Non-Judging	24.73 (6.56)	23.96 (6.64)	24.22 (6.62)	27.31 (6.09)	24.66 (6.85)	25.28 (6.77)	24.56 (5.81)	24.49 (7.28)	24.51 (6.92)	25.19 (6.29)	24.35 (6.95)	24.59 (6.78)
FFMQ Non-Reacting	18.09 (4.14)	17.02 (3.55)	17.39 (3.79)	18.72 (2.98)	17.48 (3.83)	17.77 (3.68)	17.71 (3.38)	16.86 (3.89)	17.08 (3.78)	18.09 (3.69)	17.08 (3.77)	17.37 (3.77)
ELS Recognizing Values	15.96 (2.76)	15.46 (2.80)	15.63 (2.79)	15.98 (3.06)	16.06 (2.37)	16.04 (2.54)	15.53 (2.74)	16.10 (2.95)	15.96 (2.91)	15.82 (2.81)	15.87 (2.77)	15.85 (2.78)
ELS Clarity in Life Direction	10.39 (2.95)	10.00 (2.80)	10.14 (2.86)	10.54 (2.81)	10.11 (2.61)	10.21 (2.66)	9.87 (2.95)	10.45 (2.95)	10.30 (2.95)	10.24 (2.92)	10.21 (2.82)	10.22 (2.85)
ELS Value–Action Congruence	7.61 (1.55)	7.53 (1.60)	7.56 (1.58)	7.44 (1.85)	7.82 (1.32)	7.73 (1.47)	7.55 (1.50)	7.72 (1.51)	7.67 (1.51)	7.56 (1.59)	7.68 (1.50)	7.65 (1.53)
ELS Life Fulfillment	23.96 (6.01)	23.33 (5.83)	23.55 (5.90)	25.70 (5.67)	24.18 (5.53)	24.53 (5.59)	23.25 (5.45)	23.50 (5.95)	23.43 (5.82)	24.07 (5.80)	23.62 (5.81)	23.74 (5.81)
AAQ-II	24.01 (9.38)	26.36 (9.91)	25.54 (9.78)	19.62 (8.79)	23.88 (9.33)	22.88 (9.37)	23.27 (9.32)	25.56 (9.74)	24.97 (9.68)	22.88 (9.36)	25.40 (9.73)	24.68 (9.69)

*Note*: FFMQ = Five Facet Mindfulness Scale; ELS = Engaged Living Scale; AAQ = Acceptance and Action Questionnaire.

**Table 2:** Two-way ANCOVA statistics for FFMQ scores.

	*df*	*F Ratio*	*p*	*η_p_* ^2^
**FFMQ Observing**				
Age	1	2.63	0.105	0.002
Sexual Orientation	1	16.25	<0.001	0.015
Gender	1	0.46	0.497	0.000
Race	2	7.27	<0.001	0.014
Gender × Race Interaction	2	0.690	0.502	0.001
**FFMQ Describing**				
Age	1	6.41	0.011	0.006
Sexual Orientation	1	9.09	0.003	0.009
Gender	1	5.00	0.026	0.005
Race	2	4.33	0.013	0.008
Gender × Race Interaction	2	1.50	0.225	0.003
**FFMQ Non-Distractibility**				
Age	1	3.54	0.060	0.003
Sexual Orientation	1	21.82	<0.001	0.020
Gender	1	10.48	0.001	0.010
Race	2	1.19	0.305	0.002
Gender × Race Interaction	2	2.04	0.131	0.004
**FFMQ Non-Autopiloting**				
Age	1	3.88	0.049	0.004
Sexual Orientation	1	23.02	<0.001	0.021
Gender	1	6.23	0.013	0.006
Race	2	0.10	0.902	0.000
Gender × Race Interaction	2	3.47	0.032	0.007
**FFMQ Non-Judging**				
Age	1	8.83	0.003	0.008
Sexual Orientation	1	14.14	<0.001	0.013
Gender	1	4.48	0.035	0.004
Race	2	3.57	0.029	0.007
Gender × Race Interaction	2	1.37	0.254	0.003
**FFMQ Non-Reacting**				
Age	1	2.41	0.121	0.002
Sexual Orientation	1	0.29	0.592	0.000
Gender	1	15.80	<0.001	0.015
Race	2	2.68	0.069	0.005
Gender × Race Interaction	2	0.13	0.882	0.000

*Note. N* = 1068. ANCOVA = analysis of co-variance; FFMQ = Five Facet Mindfulness Questionnaire.

**Table 3:** Two-way ANCOVA statistics for ELS scores.

	*df*	*F Ratio*	*p*	*η_p_* ^2^
**ELS Recognizing Values**				
Age	1	3.27	0.071	0.003
Sexual Orientation	1	11.98	<0.001	0.011
Gender	1	0.27	0.605	0.000
Race	2	0.79	0.456	0.001
Gender × Race Interaction	2	3.29	0.038	0.006
**ELS Clarity in Life Direction**				
Age	1	6.50	0.011	0.006
Sexual Orientation	1	3.56	0.060	0.003
Gender	1	0.04	0.842	0.000
Race	2	0.11	0.893	0.000
Gender × Race Interaction	2	2.62	0.073	0.005
**ELS Value–Action Congruence**				
Age	1	0.30	0.586	0.000
Sexual Orientation	1	4.54	0.033	0.004
Gender	1	2.56	0.110	0.002
Race	2	0.26	0.770	0.000
Gender × Race Interaction	2	1.75	0.175	0.003
**ELS Life Fulfillment**				
Age	1	0.43	0.512	0.000
Sexual Orientation	1	14.97	<0.001	0.014
Gender	1	1.72	0.190	0.002
Race	2	4.47	0.012	0.008
Gender × Race Interaction	2	1.17	0.310	0.002

*Note. N* = 1068. ANCOVA = analysis of co-variance; ELS = Engaged Living Scale.

**Table 4:** Two-way ANCOVA statistics for AAQ-II.

	*df*	*F Ratio*	*p*	*η_p_* ^2^
**AAQ-II**				
Age	1	2.47	0.120	0.002
Sexual Orientation	1	35.49	<0.001	0.032
Gender	1	15.90	<0.001	0.015
Race	2	8.31	<0.001	0.015
Gender × Race Interaction	2	0.27	0.760	0.001

*Note. N* = 1068. ANCOVA = analysis of co-variance; AAQ-II = Acceptance and Action Questionnaire-II.

**Table 5:** Means, standard deviations, coefficient alphas, and zero-order relations between all variables in the Asian American group (*n* = 402).

	1	2	3	4	5	6	7	8	9	10	11	12
1. Psychological Distress (GHQ)	--											
2. FFMQ Observing	0.13 *	--										
3. FFMQ Describing	−0.18 *	0.40 **	--									
4. FFMQ Non-Distractibility	−0.46 **	−0.28 **	0.12 *	--								
5. FFMQ Non-Autopiloting	−0.41 **	−0.20 **	0.12 *	0.69 **	--							
6. FFMQ Non-Judging	−0.45 **	−0.31 **	0.08 *	0.53 **	0.50 **	--						
7. FFMQ Non-Reacting	−0.18 **	0.39 **	0.36 **	0.07 *	−0.02	−0.02 **	--					
8. ELS Recognizing Values	−0.41 **	0.21 **	0.31 **	0.20 **	0.24 **	0.16 **	0.27 **	--				
9. ELS Clarity in Life Direction	−0.43 **	0.09	0.29 **	0.25 **	0.23 **	0.27 **	0.17 **	0.63 **	--			
10. ELS Value–Action Congruence	−0.25 **	0.12 *	0.23 **	0.20 **	0.16 **	0.08	0.23 **	0.59 **	0.44 **	--		
11. ELS Life Fulfillment	−0.54 **	0.04	0.29 **	0.33 **	0.26 **	0.31 **	0.24 **	0.58 **	0.61 **	0.53 **	--	
12. Psychological Inflexibility (AAQ-II)	0.62 **	0.15 *	−0.27 **	−0.53 **	−0.45 **	−0.62 **	−0.21 **	−0.39 **	−0.38 **	−0.25 **	−0.56 **	--
13. Age	0.04	<0.01	0.03	0.06	0.05	0.04	−0.02	0.08	−0.08	−0.01	−0.08	<−0.01
*M*	14.60	27.35	15.11	11.98	13.12	24.22	17.39	15.63	10.14	7.56	23.55	25.54
*SD*	6.64	6.18	4.13	3.68	3.47	6.62	3.79	2.79	2.86	1.58	5.90	9.78
α	0.90	0.82	0.87	0.88	0.89	0.90	0.75	0.84	0.85	0.78	0.91	0.92

* *p* < 0.05, ** *p* < 0.01. Note: GHQ = General Health Questionnaire, FFMQ = Five Facet Mindfulness Scale, ELS = Engaged Living Scale, AAQ = Acceptance and Action Questionnaire.

**Table 6:** Means, standard deviations, coefficient alphas, and zero-order relations between all variables in the White American group (*n* = 260).

	1	2	3	4	5	6	7	8	9	10	11	12
1. Psychological Distress (GHQ)	--											
2. FFMQ Observing	0.02	--										
3. FFMQ Describing	−0.34 **	0.40 **	--									
4. FFMQ Non-Distractibility	−0.44 **	−0.23 **	0.14 *	--								
5. FFMQ Non-Autopiloting	−0.48 **	−0.08	0.26 **	0.74 **	--							
6. FFMQ Non-Judging	−0.46 **	−0.16 *	0.23 **	0.47 **	0.51 **	--						
7. FFMQ Non-Reacting	−0.36 **	0.36 **	0.34 **	0.20 **	0.21 **	0.30 **	--					
8. ELS Recognizing Values	−0.39 **	0.14 *	0.35 **	0.30 **	0.29 **	0.23 **	0.26 **	--				
9. ELS Clarity in Life Direction	−0.30 **	−0.05	0.20 **	0.30 **	0.23 **	0.21 **	0.19 **	0.58 **	--			
10. ELS Value–Action Congruence	−0.36 **	0.12 *	0.30 **	0.22 **	0.29 **	0.29 **	0.28 **	0.72 **	0.44 **	--		
11. ELS Life Fulfillment	−0.53 **	0.09	0.31 **	0.41 **	0.43 **	0.41 **	0.36 **	0.65 **	0.49 **	0.70 **	--	
12. Psychological Inflexibility (AAQ-II)	0.57 **	0.08	−0.30 **	−0.54 **	−0.49 **	−0.62 **	−0.35 **	−0.42 **	−0.40 **	−0.40 **	−0.59 **	--
13. Age	−0.04	0.06	0.08	0.09	0.06	0.11	0.04	0.05	0.08	0.02	−0.01	−0.03
*M*	13.90	29.45	16.22	11.37	12.78	25.28	17.77	16.04	10.21	7.73	24.53	20.88
*SD*	6.52	6.07	4.02	3.66	3.68	6.77	3.68	2.54	2.66	1.47	5.59	9.37
α	0.89	0.82	0.87	0.89	0.87	0.92	0.75	0.82	0.82	0.78	0.90	0.93

* *p* < 0.05, ** *p* < 0.01. Note: GHQ = General Health Questionnaire, FFMQ = Five Facet Mindfulness Scale, ELS = Engaged Living Scale, AAQ = Acceptance and Action Questionnaire.

**Table 7:** Means, standard deviations, coefficient alphas, and zero-order relations between all variables in the All Others group (*n* = 406).

	1	2	3	4	5	6	7	8	9	10	11	12
1. Psychological Distress (GHQ)	--											
2. FFMQ Observing	0.10 *	--										
3. FFMQ Describing	−0.23 **	0.35 **	--									
4. FFMQ Non-Distractibility	−0.37 **	−0.11 *	0.31 **	--								
5. FFMQ Non-Autopiloting	−0.33 **	−0.06	0.25 **	0.61 **	--							
6. FFMQ Non-Judging	−0.52 **	−0.24 **	0.23 **	0.41 **	0.43 **	--						
7. FFMQ Non-Reacting	−0.27 **	0.34 **	0.38 **	0.28 **	0.17 **	0.17 **	--					
8. ELS Recognizing Values	−0.33 **	0.15 **	0.30 **	0.18 **	0.21 **	0.19 **	0.21 **	--				
9. ELS Clarity in Life Direction	−0.27 **	0.16 **	0.26 **	0.15 **	0.21 **	0.19 **	0.15 **	0.58 **	--			
10. ELS Value–Action Congruence	−0.40 **	0.08	0.30 **	0.37 **	0.33 **	0.32 **	0.22 **	0.56 **	0.42 **	--		
11. ELS Life Fulfillment	−0.54 **	0.05	0.30 **	0.28 **	0.26 **	0.44 **	0.27 **	0.59 **	0.52 **	0.63 **	--	
12. Psychological Inflexibility (AAQ-II)	0.58 **	0.11 *	−0.22 **	−0.43 **	−0.41 **	−0.58 **	−0.33 **	−0.31 **	−0.25 **	−0.39 **	−0.53 **	--
13. Age	−0.05	0.09	0.13 **	0.04	0.07	0.13 **	0.12 **	0.07	0.09	0.03	0.03	−0.10 *
*M*	14.67	28.75	15.68	11.51	12.88	24.51	17.08	15.96	10.30	7.67	23.43	24.97
*SD*	6.74	6.38	4.31	3.64	3.30	6.92	3.78	2.91	2.95	1.51	5.82	9.68
α	0.89	0.89	0.87	0.87	0.86	0.91	0.74	0.85	0.86	0.73	0.90	0.92

* *p* < 0.05, ** *p* < 0.01. Note: GHQ = General Health Questionnaire, FFMQ = Five Facet Mindfulness Scale, ELS = Engaged Living Scale, AAQ = Acceptance and Action Questionnaire.

**Table 8:** Final step of a hierarchical linear regression examining the roles of the facets of mindfulness, engaged living, and psychological inflexibility on psychological distress by racial groups.

	Asian American (n = 402)			White American (n = 260)			All Others (n = 402)		
Psychological Distress (GHQ)	*b (95% CI)*	*SE*	*β*	*b (95% CI)*	*SE*	*β*	*b (95% CI)*	*SE*	*β*
*Intercept*	20.38 (13.28, 27.48)	3.61		23.76 (15.79, 33.28)	4.44		24.02 (17.69, 30.43)	3.25	
Age	0.08 (−0.05, 0.20)	0.06	0.05	0.02 (−0.14, 0.18)	0.08	0.01	0.02 (−0.10, 0.14)	0.06	0.01
Gender	0.52 (−0.51, 1.54)	0.52	0.04	1.63 (−0.14, 2.85) *	0.78	0.11	1.02 (−0.10, 2.19)	0.59	0.07
Sexual Orientation	0.36 (−1.14, 1.86)	0.76	0.02	1.32 (−0.61, 2.79)	0.87	0.08	0.16 (−1.13, 1.49)	0.67	0.01
FFMQ Observing	0.06 (−0.03, 0.16)	0.05	0.06	0.08 (−0.05, 0.20)	0.07	0.07	0.06 (−0.04, 0.15)	0.05	0.05
FFMQ Describing	0.05 (−0.09, 0.19)	0.07	0.03	−0.20 (−0.35, 0.01) *	0.09	−0.13	−0.02 (−0.16, 0.12)	0.07	−0.01
FFMQ Non-Distractibility	−0.20 (−0.39, 0.00) *	0.10	−0.11	−0.02 (−0.25, 0.28)	0.14	−0.01	−0.12 (−0.30, 0.08)	0.10	−0.06
FFMQ Non-Autopiloting	−0.15 (−0.35, 0.04)	0.10	−0.08	−0.26 (−0.57, 0.01)	0.15	−0.13	<−0.01 (−0.20, 0.19)	0.10	<−0.01
FFMQ Non-Judging	−0.04 (−0.14, 0.06)	0.05	−0.04	−0.07 (−0.20, 0.05)	0.06	−0.08	−0.20 (−0.30, −0.10) **	0.05	−0.21
FFMQ Non-Reacting	−0.09 (−0.23, 0.06)	0.08	−0.05	−0.21 (−0.43, −0.04) *	0.10	−0.12	−0.11 (−0.27, 0.05)	0.08	−0.06
ELS Recognizing Value	−0.31 (−0.57, −0.04) *	0.14	−0.13	−0.24 (−0.65, 0.13)	0.20	−0.09	−0.11 (−0.35, 0.13)	0.12	−0.05
ELS Clarity in Life Direction	−0.21 (−0.45, 0.04)	0.12	−0.09	0.08 (−0.24, 0.33)	0.15	0.03	0.01 (−0.21, 0.22)	0.11	<0.01
ELS Value–Action Congruence	0.41 (0.02, 0.80) *	0.20	0.10	0.13 (−0.52, 0.86)	0.35	0.03	−0.16 (−0.63, 0.29)	0.24	−0.04
ELS Life Fulfillment	−0.25 (−0.37, −0.12) **	0.06	−0.22	−0.22 (−0.41, −0.04) *	0.09	−0.19	−0.27 (−0.40, −0.13) **	0.07	−0.23
Psychological Inflexibility (AAQ-II)	0.21 (0.13, 0.28) **	0.04	0.31	0.14 (0.04, 0.24) **	0.05	0.19	0.17 (0.10, 0.24) **	0.04	0.25
*Second Step R* ^2^	0.49			0.47			0.46		
*Second Step R* ^2^ *_Δ_*	0.47 **			0.34 **			0.44 **		

* *p* < 0.05, ** *p* < 0.01. Note: GHQ = General Health Questionnaire, FFMQ = Five Facet Mindfulness Scale, ELS = Engaged Living Scale, AAQ = Acceptance and Action Questionnaire.

## Data Availability

The datasets generated during and/or analyzed during the current study are available from the corresponding author on reasonable request.

## Notes

1 The language used to describe sexual orientation response options has been modified from the original survey instrument to reflect identity-affirming terminology, in accordance with APA Guidelines for Psychological Practice with Sexual Minority Persons (2021) Available online: https://www.apa.org/about/policy/psychological-sexual-minority-persons.pdf (accessed on 11 March 2026).

2 To control for Type I error inflation across the models, a Benjamini–Hochberg false discovery rate (FDR) correction was also applied across all statistical tests. All reported significant effects met the FDR-adjusted significance threshold of q < 0.05.

## References

[B1-behavsci-16-01010] Aiken L. S., West S. G. (1991). Multiple regression: Testing and interpreting interactions.

[B2-behavsci-16-01010] American Psychological Association (2015). Guidelines for psychological practice with transgender and gender nonconforming people. American Psychologist.

[B3-behavsci-16-01010] Baer R. A. (2018). Mindfulness practice. Process-based CBT: The science and core clinical competencies of cognitive behavioral therapy.

[B4-behavsci-16-01010] Baer R. A. (2019). Assessment of mindfulness by self-report. Current Opinion in Psychology.

[B5-behavsci-16-01010] Baer R. A., Smith G. T., Hopkins J., Krietemeyer J., Toney L. (2006). Using self-report assessment methods to explore facets of mindfulness. Assessment.

[B6-behavsci-16-01010] Baer R. A., Smith G. T., Lykins E., Button D., Krietemeyer J., Sauer S., Walsh E., Duggan D., Williams J. M. G. (2008). Construct validity of the five facet mindfulness questionnaire in meditating and nonmeditating samples. Assessment.

[B7-behavsci-16-01010] Bond F. W., Hayes S. C., Baer R. A., Carpenter K. M., Guenole N., Orcutt H. K., Waltz T., Zettle R. D. (2011). Preliminary psychometric properties of the acceptance and action questionnaire–II: A revised measure of psychological inflexibility and experiential avoidance. Behavior Therapy.

[B8-behavsci-16-01010] Campbell A., Vance S. R., Dong S. (2018). Examining the relationship between mindfulness and multicultural counseling competencies in counselor trainees. Mindfulness.

[B9-behavsci-16-01010] Carpenter J. K., Conroy K., Gomez A. F., Curren L. C., Hofmann S. G. (2019). The relationship between trait mindfulness and affective symptoms: A meta-analysis of the five facet mindfulness questionnaire (FFMQ). Clinical Psychology Review.

[B10-behavsci-16-01010] Cherry K. M., Hoeven E. V., Patterson T. S., Lumley M. N. (2021). Defining and measuring “psychological flexibility”: A narrative scoping review of diverse flexibility and rigidity constructs and perspectives. Clinical Psychology Review.

[B11-behavsci-16-01010] Christopher M. S., Neuser N. J., Michael P. G., Baitmangalkar A. (2012). Exploring the psychometric properties of the five facet mindfulness questionnaire. Mindfulness.

[B12-behavsci-16-01010] Cochran S. D., Sullivan J. G., Mays V. M. (2003). Prevalence of mental disorders, psychological distress, and mental health services use among lesbian, gay, and bisexual adults in the United States. Journal of Consulting and Clinical Psychology.

[B13-behavsci-16-01010] Diener E., Oishi S., Tay L. (2018). Advances in subjective well-being research. Nature Human Behaviour.

[B14-behavsci-16-01010] Di Plinio S., Northoff G., Ebisch S. J. H. (2024). The degenerate coding of psychometric profiles through functional connectivity archetypes. Frontiers in Human Neuroscience.

[B15-behavsci-16-01010] Doorley J. D., Goodman F. R., Kelso K. C., Kashdan T. B. (2020). Psychological flexibility: What we know, what we do not know, and what we think we know. Social and Personality Psychology Compass.

[B16-behavsci-16-01010] Edwards K. A., Vowles K. E. (2020). Acceptance and action questionnaire—II: Confirmatory factor analysis and measurement invariance between Non-Hispanic White and Hispanic/Latinx undergraduates. Journal of Contextual Behavioral Science.

[B17-behavsci-16-01010] Flake J. K., Pek J., Hehman E. (2017). Construct validation in social and personality research: Current practice and recommendations. Social Psychological and Personality Science.

[B18-behavsci-16-01010] Fledderus M., Oude Voshaar M. A. H., ten Klooster P. M., Bohlmeijer E. T. (2012). Further evaluation of the psychometric properties of the acceptance and action questionnaire–II. Psychological Assessment.

[B19-behavsci-16-01010] Goldberg D. P., Gater R., Sartorius N., Ustun T. B. (1997). The validity of two versions of the GHQ in the WHO study of mental illness in general health care. Psychological Medicine: A Journal of Research in Psychiatry and the Allied Sciences.

[B20-behavsci-16-01010] Hayes S. C., Follette V. M., Linehan M. M. (2004). Mindfulness and acceptance: Expanding the cognitive-behavioral tradition.

[B21-behavsci-16-01010] Hayes S. C., Pistorello J., Levin M. E. (2012a). Acceptance and commitment therapy as a unified model of behavior change. The Counseling Psychologist.

[B22-behavsci-16-01010] Hayes S. C., Strosahl K. D., Wilson K. G. (2012b). Acceptance and commitment therapy: The process and practice of mindful change.

[B23-behavsci-16-01010] Hayes S. C., Villatte M., Levin M., Hildebrandt M. (2011). Open, aware, and active: Contextual approaches as an emerging trend in the behavioral and cognitive therapies. Annual Review of Clinical Psychology.

[B24-behavsci-16-01010] Haynes S. N., Kaholokula J. K. A., Tanaka-Matsumi J. (2018). Psychometric foundations of psychological assessment with diverse cultures: What are the concepts, methods, and evidence?. Cultural competence in applied psychology.

[B25-behavsci-16-01010] Haynes S. N., Smith G. T., Hunsley J. D. (2019). Scientific foundations of clinical assessment.

[B26-behavsci-16-01010] Hays P. A. (2009). Integrating evidence-based practice, cognitive–behavior therapy, and multicultural therapy: Ten steps for culturally competent practice. Professional Psychology: Research and Practice.

[B27-behavsci-16-01010] Hilert A. J., Tirado C. (2019). Teaching multicultural counseling with mindfulness: A contemplative pedagogy approach. International Journal for the Advancement of Counselling.

[B28-behavsci-16-01010] Holland A. T., Palaniappan L. P. (2012). Problems with the collection and interpretation of Asian-American health data: Omission, aggregation, and extrapolation. Annals of Epidemiology.

[B29-behavsci-16-01010] Jo D., Im S., Suh D. E., Spencer S. D., Masuda A. (2023). The personalized psychological flexibility index (PPFI): An item response theory analysis with racially diverse college students. Journal of Psychopathology and Behavioral Assessment.

[B30-behavsci-16-01010] Jo D., Pan M. C., Masuda A. (2025). Understanding mindfulness in racially diverse adults through a person-oriented approach: A latent profile analysis with a Hawaiʻi-based sample. Mindfulness.

[B31-behavsci-16-01010] Jo D., Spencer S. D., Masuda A. (2022). Mindfulness as a moderator of the relationship between engaged living and depression in emerging adulthood. Mindfulness.

[B32-behavsci-16-01010] Johnson D. P., Whisman M. A. (2013). Gender differences in rumination: A meta-analysis. Personality and Individual Differences.

[B33-behavsci-16-01010] Kam B., Mendoza H., Masuda A. (2019). Mental health help-seeking experience and attitudes in Latina/o American, Asian American, Black American, and White American college students. International Journal for the Advancement of Counselling.

[B34-behavsci-16-01010] Kim B. S. K., Atkinson D. R., Umemoto D. (2001). Asian cultural values and the counseling process: Current knowledge and directions for future research. The Counseling Psychologist.

[B35-behavsci-16-01010] Komiya N., Good G. E., Sherrod N. B. (2000). Emotional openness as a predictor of college students’ attitudes toward seeking psychological help. Journal of Counseling Psychology.

[B36-behavsci-16-01010] Lin S. L., Jo D., Spencer S. D., Masuda A. (2024). The role of engaged living in the association between self-concealment and psychological distress among racially diverse college students in Hawaiʻi. Journal of Contextual Behavioral Science.

[B37-behavsci-16-01010] Masuda A. (2014). Mindfulness and acceptance in multicultural competency: A contextual approach to sociocultural diversity in theory and practice.

[B38-behavsci-16-01010] Masuda A., Qinaʻau J., Juberg M., Martin T., Benuto L. T., Duckworth M. P., Masuda A., O’Donohue W. (2020). Bias in the diagnostic and statistical manual 5 and psychopathology. Prejudice, stigma, privilege, and oppression: A behavioral health handbook.

[B39-behavsci-16-01010] Meehl P. E. (1978). Theoretical risks and tabular asterisks: Sir Karl, Sir Ronald, and the slow progress of soft psychology. Journal of Consulting and Clinical Psychology.

[B40-behavsci-16-01010] Molenaar P. C. M. (2004). A manifesto on psychology as idiographic science: Bringing the person back into scientific psychology, this time forever. Measurement: Interdisciplinary Research and Perspectives.

[B41-behavsci-16-01010] Molina J. G., Rodrigo M. F., Losilla J.-M., Vives J. (2014). Wording effects and the factor structure of the 12-item General Health Questionnaire (GHQ-12). Psychological Assessment.

[B42-behavsci-16-01010] Morgan J. R., Masuda A., Anderson P. L. (2014). A preliminary analysis of the psychometric properties of the mindful attention awareness scale among African American college students. Mindfulness.

[B43-behavsci-16-01010] Nakamura L., Jo D., Masuda A. (2022). Mental health help-seeking experience and attitudes in Asian American, Multiracial American, and White American emerging adults. International Journal for the Advancement of Counselling.

[B44-behavsci-16-01010] Oishi S., Diener E., Lucas R. E., Suh E. M., Diener E. (2009). Cross-cultural variations in predictors of life satisfaction: Perspectives from needs and values. Culture and well-being: The collected works of ed diener.

[B45-behavsci-16-01010] Park Y. S., Kim B. S. K. (2008). Asian and European American cultural values and communication styles among Asian American and European American college students. Cultural Diversity and Ethnic Minority Psychology.

[B46-behavsci-16-01010] Pitman A., Marston L., Lewis G., Semlyen J., McManus S., King M. (2022). The mental health of lesbian, gay, and bisexual adults compared with heterosexual adults: Results of two nationally representative English household probability samples. Psychological Medicine.

[B47-behavsci-16-01010] Putnick D. L., Bornstein M. H. (2016). Measurement invariance conventions and reporting: The state of the art and future directions for psychological research. Developmental Review.

[B48-behavsci-16-01010] Rochefort C., Baldwin A. S., Chmielewski M. (2018). Experiential avoidance: An examination of the construct validity of the AAQ-II and MEAQ. Behavior Therapy.

[B49-behavsci-16-01010] Sasa S. M., Yellow Horse A. J. (2022). Just data representation for Native Hawaiians and Pacific Islanders: A critical review of systemic Indigenous erasure in census and recommendations for psychologists. American Journal of Community Psychology.

[B50-behavsci-16-01010] Schwartz S. H., Rubel-Lifschitz T. (2009). Cross-national variation in the size of sex differences in values: Effects of gender equality.

[B51-behavsci-16-01010] Segal Z. V., Williams J. M. G., Teasdale J. D. (2013). Mindfulness-based cognitive therapy for depression.

[B52-behavsci-16-01010] Spencer S. D., Jo D., Hamagami F., Antonio M. C. K., Qinaʻau J., Masuda A., Hishinuma E. S. (2022). A psychometric validation of contextual cognitive behavioral therapy-informed measures with racially and ethnically diverse adults. Journal of Contextual Behavioral Science.

[B53-behavsci-16-01010] Spencer S. D., Tyndall I., Levin M. E., Twohig M. P. (in press). Assessment of psychological flexibility: Conceptual foundations, empirical support, and clinical applications. Optimizing ACT.

[B54-behavsci-16-01010] Trompetter H. R., ten Klooster P. M., Schreurs K. M. G., Fledderus M., Westerhof G. J., Bohlmeijer E. T. (2013). Measuring values and committed action with the engaged living scale (ELS): Psychometric evaluation in a nonclinical sample and a chronic pain sample. Psychological Assessment.

[B55-behavsci-16-01010] Tyndall I., Waldeck D., Pancani L., Whelan R., Roche B., Dawson D. L. (2019). The acceptance and action questionnaire-II (AAQ-II) as a measure of experiential avoidance: Concerns over discriminant validity. Journal of Contextual Behavioral Science.

[B56-behavsci-16-01010] U.S. Census Bureau (2019). Population estimates, July 1, 2019 (V2019)—Hawaii. Quick facts.

[B57-behavsci-16-01010] van de Vijver F., Tanzer N. K. (2004). Bias and equivalence in cross-cultural assessment: An overview. European Review of Applied Psychology.

[B58-behavsci-16-01010] Washburn J. J., Teachman B. A., Gaudiano B. A., Penberthy K., Peris T. S., Davison G. C., Hollon S. D. (2022). The central role of lifelong learning and humility in clinical psychology. Clinical Psychological Science.

[B59-behavsci-16-01010] Williams M. J., Dalgleish T., Karl A., Kuyken W. (2014). Examining the factor structures of the five facet mindfulness questionnaire and the self-compassion scale. Psychological Assessment.

[B60-behavsci-16-01010] Wolgast M. (2014). What does the acceptance and action questionnaire (AAQ-II) really measure?. Behavior Therapy.

[B61-behavsci-16-01010] Yarnell L. M., Neff K. D., Davidson O. A., Mullarkey M. (2019). Gender differences in self-compassion: Examining the role of gender role orientation. Mindfulness.

